# S82. GOAL MANAGEMENT TRAINING OF EXECUTIVE FUNCTIONS FOR PATIENTS WITH SCHIZOPHRENIA OR HIGH RISK OF SCHIZOPHRENIA: BASELINE CHARACTERISTICS AND PRELIMINARY RESULTS FROM AN RCT

**DOI:** 10.1093/schbul/sby018.869

**Published:** 2018-04-01

**Authors:** Ingvild Haugen, Elisabeth Haug, Torill Ueland, Jan Stubberud, Merete Øie

**Affiliations:** 1 Innlandet Hospital Trust; 2 Oslo University Hospital; 3 Lovisenberg Diaconal Hospital, University of Oslo

## Abstract

**Background:**

About 85% of patients with schizophrenia have cognitive impairments, executive functions being particularly affected. Executive dysfunctions are important predictors of functional outcomes. Unlike psychotic symptoms, cognitive deficits do not improve during periods of remission and change only minimally with antipsychotic medications. Thus, effective interventions aimed at improving executive functions in this population are needed.

One of the most validated interventions for executive dysfunction is Goal Management Training (GMT). GMT is a compensatory intervention that relies on metacognitive strategies for improving participants’ ability to organize and achieve goals in everyday life. GMT has received empirical support in studies of other populations, such as people with neurological conditions and in healthy elderly. To our knowledge no previous studies have investigated the effect of group-based GMT in patients with schizophrenia spectrum disorders or with high risk of schizophrenia. Thus, this is the main objective of the study. Baseline characteristis and preliminary results from the first patients will be presented.

**Methods:**

Participants (16–67 years, males and females, IQ >70) with executive dysfunction, will be recruited among patients referred for treatment at Innlandet Hospital Trust in Norway from 2017 to 2020. The study aims to include patients treated for psychotic disorder for less than 5 years and new patients who either have symptoms that meet the DSM-IV criteria for a diagnosis of broad schizophrenia spectrum disorder or who are considered at high risk of psychosis. We aim to recruit one hundred participants for the current randomized controlled trial (RCT), with efficacy of GMT (n = 50) being compared with results of subjects in a wait-list condition (WL, n = 50).

Measurements include self-report of executive function, emotional health, and social- and everyday function. Informant reports of executive function will also be collected. Furthermore, neuropsychological tests designed specifically to measure areas of executive function will be utilized, as well as role-playing tasks thought to have good ecological validity. Symptoms of psychosis will also be assessed. GMT will be administered in 9 (twice weekly) x 2 hour sessions in accordance with the GMT research protocol.

A general linear model with repeated measures analysis of variance (RM ANOVA) will be used to examine differential group treatment effects. A 2 x 3 mixed-design will be applied, with Group (GMT, WL) as between-subjects factor, and Session (baseline [T1], post-intervention [T2], and 6 months follow-up [T3]) as within-subjects factor. Interpretation of the strength of experimental effects will be provided with effect size statistics.

**Results:**

Baseline characteristics and preliminary results from the first participants will be presented.

**Discussion:**

Based on findings from previous GMT-studies, we hypothesize that post-intervention changes will be reflected in improved scores on self-reported and/or objective measures of executive functions (particularly in the areas of planning and attentional control) compared to patients in WL. We also expect that GMT participants will improve their goal attainment in everyday life and social functioning after the intervention. Additionally, we expect post-intervention changes to be reflected in improved scores on measures of emotional health.

